# Emerging roles for microtubules in angiosperm pollen tube growth highlight new research cues

**DOI:** 10.3389/fpls.2015.00051

**Published:** 2015-02-10

**Authors:** Elisabetta Onelli, Aurora I. Idilli, Alessandra Moscatelli

**Affiliations:** ^1^Department of Biosciences, University of Milan, Milan, Italy; ^2^Institute of Biophysics, National Research Council and Fondazione Bruno Kessler, Trento, Italy

**Keywords:** pollen tube, microtubules, polarized growth, trafficking, cell wall, pollen tube-pistil crosstalk

## Abstract

In plants, actin filaments have an important role in organelle movement and cytoplasmic streaming. Otherwise microtubules (MTs) have a role in restricting organelles to specific areas of the cell and in maintaining organelle morphology. In somatic plant cells, MTs also participate in cell division and morphogenesis, allowing cells to take their definitive shape in order to perform specific functions. In the latter case, MTs influence assembly of the cell wall, controlling the delivery of enzymes involved in cellulose synthesis and of wall modulation material to the proper sites. In angiosperm pollen tubes, organelle movement is generally attributed to the acto-myosin system, the main role of which is in distributing organelles in the cytoplasm and in carrying secretory vesicles to the apex for polarized growth. Recent data on membrane trafficking suggests a role of MTs in fine delivery and repositioning of vesicles to sustain pollen tube growth. This review examines the role of MTs in secretion and endocytosis, highlighting new research cues regarding cell wall construction and pollen tube-pistil crosstalk, that help unravel the role of MTs in polarized growth.

## INTRODUCTION

In plant cells, microtubules (MTs) play crucial roles in cell division, expansion and morphogenesis. Unlike in animals, cytoplasmic streaming and organelle movement in plant cells are mostly attributed to the actin cytoskeleton (Shimmen, 2007). The specific role of actin filaments is thought to have been inherited by land plants from an ancestral streptophyte algae; in fact, in the giant internodal cells of Characean algae, myosin generates very fast cytoplasmic streaming which depends on a dramatic acceleration of ADP release and an extremely fast ATP binding rate (Ito et al., 2003, 2007). Thus myosin motors enable more efficient organelle and metabolite movement in large plant cells. The recent use of high- and low-speed chimeric myosin expressed in *Arabidopsis thaliana* has shown that the increase and decrease in plant cell size are related to acceleration and deceleration of cytoplasmic streaming, respectively. These findings suggest that cytoplasmic streaming is one of the key regulators of plant cell size (Tominaga et al., 2013).

Although some MT-associated motor proteins, such as kinesins, seem to target and to be involved in the fine positioning of organelles at their cellular destinations, it is uncertain whether MTs participate in long-distance trafficking of organelles or vesicles (Cai et al., 2000; Cai and Cresti, 2012). The characterization of CLASP, which functions as a plus-end-tracking MT-associated protein, suggests that MTs bind to endosomes and are involved in endosome sorting and trafficking (Ambrose et al., 2013). Furthermore, CLASP mediates the interaction between MTs and plasma membrane (PM) and plays a role in the organization of MTs in the cell cortex (Ambrose et al., 2011). Thus, while actin-dependent cytoplasmic streaming uniformly redistributes organelles in plant cells, restriction of organelles to specific cell areas appears to be actin-independent and suggests the involvement of MTs (for reviews, see Brandizzi and Wasteneys, 2013; Cai et al., 2014). In addition, the maintenance of Golgi morphology, characterized by the presence of structurally and functionally independent stacks, has been associated with MT integrity (Wang et al., 2012b). It was also recently reported that MTs contribute to ER tubule elongation and anchoring in *Arabidopsis* (Hamada et al., 2014). MTs could therefore have a role in controlling organelle zonation and shape in plant cells.

During cell division, MTs arrange into the pre-prophase band, mitotic spindle and phragmoplast (for a review, see Rasmussen et al., 2013). After cell division, cells grow and take specific shapes depending on their differentiation pattern. Cell shape is determined by different mechanisms involving turgor pressure and cell wall tension and structure. The orientation of cellulose microfibrils in the cell wall has a major role in determining cell shape, and is in turn controlled by cortical MTs. A protein complex containing multiple isoforms of cellulose synthase (CESA), namely cellulose synthase complex (CSC), is located in the PM and synthesizes the glucan chains that form elementary cellulose fibrils. Elementary fibrils contain 18–24 glucan chains, suggesting that some of the CESA proteins in CSC could be enzymically inactive or that a single glucan chain could be synthesized by more that one CESA protein (Fernandes et al., 2011; Newman et al., 2013; Thomas et al., 2013; Li et al., 2014). The distribution of CSCs on the cell surface depends on correct targeting of CSC-containing vesicles to the PM. Secretion of CSC vesicles by the *trans*-Golgi network (TGN) appears to be actin-dependent, while cortical MTs play a role in positioning CESA in the appropriate PM sites (Gutierrez et al., 2009). CSC-containing Golgi bodies show a MT-dependent pause under the PM, allowing delivery of CSC vesicles to their PM sites (Crowell et al., 2009). It has also been postulated that CSC internalization and recycling are MT-dependent (Crowell et al., 2009; Bashline et al., 2014a,b).

Despite increasing knowledge of the role of MTs in membrane trafficking and in somatic cell division, growth and morphogenesis very little is known about their role in cells with polarized growth, such as pollen tube cells. In lower plants MTs play a major role in fertilization, as plants deliver flagellated male gametes that swim in an aqueous medium to reach the female gamete. In Coniferophyta, Gnetophyta, and Magnoliophyta, male gametophytes evolved the pollen tube as a biological channel to convey sperm cells to the egg cell. This new structure implied the loss of flagella; sperm cells retain a basket-like MT apparatus and a tail where MTs are crossbridged but not organized to form an axonemal structure (Cresti et al., 1990; Southworth and Cresti, 1997). The mechanism of generative/sperm cell movement along the growing pollen tube and how this movement is regulated is still being studied. It is reported that MTs control the distance of male germ units from the pollen tube tip (Laitiainen et al., 2002; Sanati Nezhad et al., 2013).

This review focuses on emerging new roles of MTs in the growth of angiosperm pollen tubes and their interactions with style tissues. Pollen tube elongation depends on polarized secretion of cell wall material and new segments of PM in a restricted area of the apex (for a recent review of the actin-mediated trafficking in the clear zone, see Hepler and Winship, 2015). The pollen tube is also characterized by an unequal distribution of proteins and lipids along the PM, made possible by selective internalization and recycling of PM domains (Moscatelli and Idilli, 2009; Onelli and Moscatelli, 2013; Hepler and Winship, 2015). The actin cytoskeleton is involved in cytoplasmic streaming and in delivering secretory vesicles to the apical PM (Hepler et al., 2001; Lovy-Wheeler et al., 2007; Cheung and Wu, 2008; Chebli et al., 2013a; Rounds et al., 2014). On the contrary, the role of MTs in polarized cell growth has not been defined. Nevertheless, new evidence suggests that MTs could be involved in short-lived movement of membranous organelles in pollen tubes (Romagnoli et al., 2003; Idilli et al., 2013).

Here we look at new evidence suggesting involvement of MTs in membrane trafficking with the aim of highlighting new cues for unraveling the structural constraints of polarized growth.

## MICROTUBULES AND VESICLE/ENDOSOME TRAFFICKING

**FIGURE 1:**
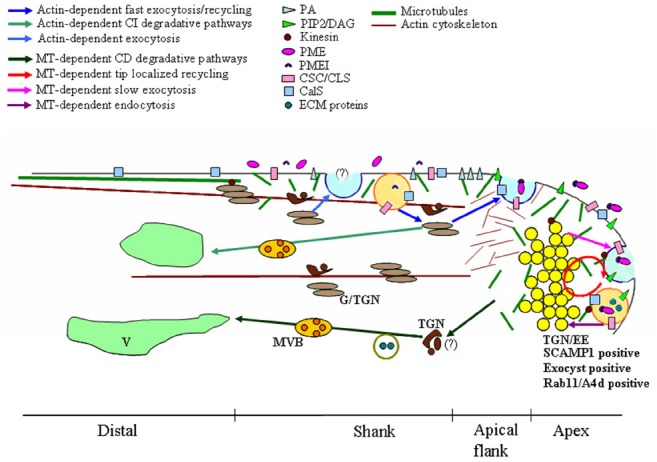
**Model of microtubules (MT)-dependent exocytic/endocytic pathways describing putative relationships between MTs, vesicles, plasma membrane (PM), endosomes and Golgi/Trans Golgi Network (G/TGN).** Dynamic MTs are organized in short randomly oriented strands in the shank and in the tip. More stable MTs form long, longitudinally oriented bundles in the cortical region of the distal area. Distinct sites of exocytosis and endocytosis occur in growing pollen tubes. The fast exocytosis (blue vesicle) and the endocytosis (orange vesicle)/recycling in the shank are both actin-dependent (blue and light blue arrows). The delivery toward the vacuole (V) of material internalized in the shank is also actin-dependent and involves both Golgi/TGN (light brown/brown compartments respectively) and (MVBs; light green arrows). In the central domain of the tip, endocytosis, recycling (red circle) and slow exocytosis (blue vesicle) are MT-dependent (plum, red, and lilac arrows respectively). Tip localized processes (endocytosis, exocytosis, and recycling) involve the clear zone, a compartment acting as an Trans-Golgi Network/early endosome (TGN/EE) and positive to SCAMP1, exocyst and Rab11/A4d (yellow vesicles). Most endocytic vesicles (orange vesicle) are delivered from this putative TGN/EE compartment to a MT-dependent degradation pathway which does not involve the Golgi apparatus (green arrows). Kinesin could also play a role in organelle movement, in maintaining the structure of Golgi stacks and in cell wall deposition. These membrane compartments are involved in cell wall building and modulation, in recycling/repositioning of proteins/lipids to maintain PM polarity and in the crosstalk between pollen tube and pistil ECM. PA, phosphatidic acid; PIP_2_, inositol 4,5-bisphosphate; DAG, diacyl glycerol; PME, pectin methylesterases; PMEI, PME inhibitor; CSC, cellulose synthase complex; CLS, cellulose-synthase-like proteins; CalS, Callose synthase; ECM proteins, pistil-secreted extracellular matrix including stigma/stylar cysteine-rich adhesin (SCA), C2 domain-containing protein (NaPCCP) and S-RNase.

A recent model of pollen tube growth postulates the presence of distinct sites of exocytosis and endocytosis (Figure 1). Fast exocytosis in the apical flanks is supposed to provide new PM and cell wall material supporting tip growth (Zonia and Munnik, 2008; Chebli et al., 2012; Moscatelli et al., 2012). This fast secretion appears to be actin-dependent while slower actin-independent exocytosis occurs in the central domain of the apex (Moscatelli et al., 2012; Rounds et al., 2014; Hepler and Winship, 2015). The excess PM secreted in the apical flanks is retrieved by an actin-dependent internalization process in the shank of the tube (Moscatelli et al., 2007, 2012; Zonia and Munnik, 2008). These endocytic vesicles are mostly recycled to the secretory pathway through Golgi apparatus and are partially destined for degradation. On the contrary, PM internalized in the apex is mostly conveyed to the degradation pathway and does not involve the Golgi apparatus. Endocytosis dissection experiments using charged nanogold have also shown a mechanism of tip-localized membrane recycling that is probably involved in regulating apical PM protein/lipid composition (Parton et al., 2001; Helling et al., 2006; Moscatelli et al., 2007).

### MICROTUBULES AFFECTED MEMBRANE DYNAMICS IN THE APEX

It was recently shown that actin-independent internalization and exocytic events taking place in the apex require MTs (Idilli et al., 2013). In *Nicotiana tabacum* pollen tube, dynamic MTs are organized in short randomly oriented strands in the shank and apex (5–30 μm from tip), whereas more stable MTs forming long, longitudinally oriented bundles are present in the cortical region of the distal area (Figure 1; Cheung et al., 2008; Idilli et al., 2012, 2013).

The presence of dynamic MTs in the shank and tip is suggested by their higher sensitivity to low concentrations of nocodazole (Noc), which does not affect MT bundles in the distal part of the tube (Idilli et al., 2013). In addition, a centrosomal homolog polypeptide, recognized by anti-6C6 monoclonal antibody, localizes in the pollen tube apex and shank PM, suggesting the presence of putative cortical MT nucleation sites in this area (Cai et al., 1996). More recently, treatment of elongating pollen tubes with plus-end-tracking MT-binding GFP-AtEB1 fusion protein revealed short dynamic MTs in the subapical region, up to 50–60 μm from the apex. These MTs entered the apical dome and localized in the core cytoplasm (Cheung et al., 2008). While undergoing changes in length, subapical MTs stably associated with the cortex, as they remained in approximately the same location over time (Cheung et al., 2008).

In somatic cells, MT rearrangement is regulated by phospholipase D (PLD), an enzyme that hydrolyzes structural phospholipids to phosphatidic acid (PA; Pleskot et al., 2013). The presence of PLD-dependent PA in the PM, due to salt-stress activation of PLD, modulates the function of MT-interacting protein MAP65 (Zhang et al., 2012) which stabilizes and bundles MTs (Smertenko et al., 2004; Lucas et al., 2011). In tobacco pollen tubes, PA localizes in the subapical region where dynamic MTs are attached to the PM through direct interaction with members of the PLDβ/δ protein subfamily; on the contrary, PA is not present in the tip where MTs enter the core cytoplasm (Potocký et al., 2003).

In more distal areas, GFP-AtEB1-labeled cables are prevalently along the cortex and appear immobile, suggesting that they represent a more stable MT population (Cheung et al., 2008).

The use of low concentrations of the MT-depolymerising agent, Noc, which affects more dynamic MTs, showed that only slow exocytosis in the central part of the tip is affected by perturbation of MTs (Idilli et al., 2013). With regard to endocytosis, MT depolymerization inhibits PM internalization in the tip and affects the sorting of tip-internalized vesicles (Figure 1).

In somatic plant cells, the TGN was identified as the early endosome (EE), the first station from which internalized material is sorted to the recycling or degradation pathway (Lam et al., 2007; Viotti et al., 2010). This compartment is defined by the presence of SCAMPs which belong to a group of transmembrane proteins playing a role in vesicle trafficking between the Golgi apparatus and PM in higher eukaryotic cells (Castle and Castle, 2005). SCAMP 1 and SCAMP 2 are localized in TGN/EEs (Lam et al., 2007; Toyooka and Matsuoka, 2009) and SCAMP 1-positive organelles are the first compartment reached by the endocytic probes FM4-64 in plant somatic cells (Lam et al., 2007). In lily pollen tubes, SCAMP1 is mainly concentrated in the apical inverted-cone region, suggesting the presence of a compartment acting as an TGN/EE in the clear zone (Figure 1; Wang et al., 2010).

In tobacco pollen tubes, acidic vesicles concentrated in the clear zone have been revealed by Lysosensor, confirming that at least part of these vesicles could represent an EE-like compartment (Moscatelli et al., 2007; Idilli et al., 2013). The movement of newly tip-internalized vesicles is delayed in the acidic inverted cone region and vesicles assigned to the degradation pathway are misallocated to the Golgi apparatus in the presence of Noc (Idilli et al., 2013).

Thus, although mathematical models show that movement of vesicles in the clear zone is governed by diffusion and advection (Kroeger et al., 2009; Chebli et al., 2013b), MTs may represent tracks for endocytic vesicles directed to the degradation pathway or they could play a role in redistribution/recycling of PM components among different membrane domains in order to maintain functional districts in the apical PM. The analysis of structure and dynamic properties of MTs in growing pollen tubes could provide major evidence of the involvement of MTs in tip-focused trafficking.

The exocyst is an important complex observed in the pollen tube tip. It is an octameric protein complex (Sec3, Sec5, Sec6, Sec8, Sec10, Sec15, Exo70, and Exo84) that plays different roles in cell growth, morphogenesis and pathogen response and tethers exocytic vesicles to the PM (Zárský et al., 2009; Cvrcková et al., 2012; Fendrych et al., 2013). It localizes exocytic vesicles in specific PM domains (Hàla et al., 2008). Zárský and colleagues propose that in plant cells exocytosis occurs mostly in exo/endocytotically active PM domains (activated cortical domain, ACD) in which exocyst complex, Rop-GTPase and lipids define PM sites for exocytosis (Zárský et al., 2009; Synek et al., 2014).

It could be interesting to investigate whether short MT strands in the tip and shank are involved in delivering vesicles to the proper exocyst-primed PM sites. The mechanism of MT-based transport could also be investigated: MTs may function as tracks for vesicle movement or MT dynamics could be the driving force of vesicles, as observed for CESA-containing vesicles during cell wall assembly (Crowell et al., 2009; Gutierrez et al., 2009).

The altered dynamics of different exocyst subunits at the PM after prolonged actin perturbation has been observed in *Arabidopsis thaliana* somatic cells, and it was hypothesized to depend on cooperation between actin filaments and MTs in defining ACDs in the PM (Fendrych et al., 2013). In the pollen tube, studies using actin filaments and MT depolymerizing drugs showed that both cytoskeletal systems cooperate in regulating membrane trafficking in the apex. However, the extent and mechanisms of interaction still need to be investigated.

In somatic plant cells, exocyst complexes are also involved in membrane recycling: in *Arabidopsis* root, the mutant of the *EXO70A1* (*exo70A1*) subunit showed defects in recycling PM proteins such as PIN1 and PIN2 (Zárský et al., 2009, 2013; Zárský and Potocký, 2010; Drdová et al., 2013). Mutants of different subunits of the exocyst complex also showed defective pollen germination and pollen tube growth. Interestingly, the components of the complex colocalized in the tobacco pollen tube tip (Cole et al., 2005; Hàla et al., 2008) where a putative early-sorting compartment involved in apical membrane recycling was described (Figure 1; Moscatelli et al., 2007; Idilli et al., 2013). Membrane recycling is considered to be a mechanism that allows the polarization of PM components to be maintained in the cell. It has been hypothesized that recycling compartments/vesicles and ACDs may be dynamic membrane compartments/superstructures called *recycling domain* (RDs; Zárský et al., 2009). Intriguingly, the existence of multiple RDs in a single plant cell could imply the presence of TGN/EE subtypes (Zárský et al., 2009) and it could be interesting to investigate whether the EE-like compartment observed in the clear zone, affected by MT depolymerising agents, might belong to a RD involved in tip polarized growth.

### MICROTUBULE MOTOR PROTEINS IN POLLEN TUBE GROWTH

Interaction between MTs and vesicles in the clear zone has been supported by detection of a kinesin-like protein that associates with these compartments aligned along apical MTs (Tiezzi et al., 1992; Cai et al., 1993; Terasaka and Niitsu, 1994). Kinesins are MT-based motor proteins which have been classified in 14 families with different roles in MT/organelle interaction. They can move to the plus or minus end of MTs and play a role in organelle movement, in maintaining the structure of Golgi stacks, in formation of Golgi-derived vesicles, in cell wall deposition and in cell division (Figure 1; for reviews, see Li et al., 2012; Zhu and Dixit, 2012). In *Corylus avellana* pollen, a kinesin-like protein has been associated with Golgi membranes and assumed to mediate their binding to MTs (Figure 1; Liu et al., 1994). It is hypothesized that MTs and kinesins are involved in the final positioning of Golgi stacks following long range transportation events mediated by the acto-myosin system (Romagnoli et al., 2007). Recent *in vitro* motility assays show that organelles of the microsomal fraction of tobacco pollen tubes move along MTs in an ATP-dependent manner (Romagnoli et al., 2003). Organelle movement occurs without cytosol and only in the presence of ATP, suggesting that functional motor proteins are stably attached to the organelle surface and that these proteins could be so far unidentified kinesins (Romagnoli et al., 2003).

The role of kinesins in the tip growth seems to be evolutionarily conserved in plants despite different models of elongation. In *Physcomitrella patens*, KINID1 kinesin binds MT plus end and controls MT dynamics in the apex of caulonemal cells. As a consequence, KINID1 participates in maintaining a thick MT bundle in the apex, thus promoting tip growth and regulating growth direction (Hiwatashi et al., 2014). Kinesins also support tip growth in root hairs and conifer pollen tubes, by delivering different cargoes along MTs to the elongating areas (Yang et al., 2007; Lazzaro et al., 2013). *Picea abies* pollen, showed a calmodulin-binding kinesin which accumulates in the elongating tip and is involved in the functional interplay between MTs and actin filaments (Lazzaro et al., 2013).

In tobacco pollen tubes, biochemical and molecular studies also show the presence of dynein-related polypeptides, the major minus-end-directed MT motor (Moscatelli et al., 1995; Scali et al., 2003; Shanina et al., 2009). Dynein-related polypeptides are present in the soluble fraction or associated with membranous organelles/vesicles uniformly distributed in the pollen tube cytoplasm (Moscatelli et al., 1995, 1998). Nevertheless, the presence of dyneins in higher plants is debated since dynein and dynactin sequences have not been identified in the *Arabidopsis* genome (Wickstead and Gull, 2007; Hammesfahr and Kollmar, 2012).

Growing pollen tubes in stiffened artificial media allowed to dissect the role of the cytoskeleton in the mechanics of pollen tube elongation: whereas actin filaments generate the force for the style tissue penetration, MTs are involved in the control of the growth direction (Gossot and Geitmann, 2007). The contribution of MTs in membrane trafficking at the tip and in repositioning of signaling protein/lipids among different regions of the apical PM, could confer to pollen tubes the ability to turn in the presence of mechanical obstacles during the route toward the embryo sac.

The possible role of MTs in membrane trafficking implies that they could be cytoplasmic determinants which modulate PM protein/lipid remodeling, cell wall synthesis and composition, and which contribute to signaling processes occurring during pollen tube elongation into pistil tissues.

## MICROTUBULES AND CELL WALL DEPOSITION

The composition of the cell wall and the spatial distribution of its components are crucial for pollen tube shaping and growth (Geitmann, 2010; Chebli et al., 2013a,b; Nezhad et al., 2013; Bashline et al., 2014b). In plants, somatic cell shape is mainly determined by the orientation of cellulose fibrils that constrain cells to expand in certain directions. The chemical configuration of pectins can also affect the mechanical properties of the cell wall (Parre and Geitmann, 2005a; Palin and Geitmann, 2012). During differentiation, plant cell volume increases considerably, allowing cells to take their final shape. This process requires an increase in cell surface area which occurs by secretion of cell wall material and simultaneous insertion of new PM tracts. It has also been hypothesized that besides being the driving force of cell expansion, turgor pressure also provides the physical strength to incorporate new cell wall material secreted by Golgi-derived vesicles (Ray, 1992; Proseus and Boyer, 2005; Hepler et al., 2013).

### PECTIN SECRETION

The chemical composition of pectins inserted into the cell wall seems to influence cell expansion. In root meristem and the elongation zone, cell growth is enabled by methyl-esterified pectins, while non-growing cells (quiescent center) show de-esterified pectins (Dolan et al., 1997). Pectins with a low degree of methyl esterification can be crosslinked by Ca^2+^ ions and this process increases the rigidity of the cell wall matrix (Cosgrove, 2005).

In angiosperm pollen tubes, the tip area undergoes cell expansion, while the shank and distal areas do not elongate. The distribution of cell wall components is consistent with this model of polarized growth (Geitmann, 2010; Chebli et al., 2013a,b). Pollen tube walls consist mainly of pectins which allow elongation at the tip and stabilize pollen tube diameter in the distal region. The very few cellulose microfibrils are mainly distributed in the sub-apex (Schlupmann et al., 1994; Taylor et al., 2003). Otherwise, callose is never observed in the tip but distributes in the sub-apical/distal regions of growing pollen tubes (Ferguson et al., 1998; Fayant et al., 2010; Cai et al., 2011; Chebli et al., 2012). The deposition of new cell wall material occurs mostly at the pollen tube tip: Golgi-derived vesicles containing highly methyl-esterified pectins are delivered to the apical flanks (Figure 1). Pectins are secreted together with pectin methylesterases (PMEs) which remove the methyl groups, allowing pectin gelation and cell wall stiffening (Willats et al., 2001). The activity of PMEs is in turn regulated by a PME inhibitor (PMEI), cosecreted with PMEs (Figure 1; Rockel et al., 2008). PMEI is observed in the tip and never in the shank cell wall, suggesting that PMEI inhibits pectin de-esterification, inducing local cell wall softening and allowing tip growth. On the contrary, PME activity in the shank stiffens the cell wall and prevents lateral cell expansion (Figure 1). The distribution of de-esterified pectins is important for maintaining pollen tube diameter and cylindrical shape. Studies performed in lily pollen tubes support a mechanical model by which the change from a high to a low degree of pectin methyl-esterification, occurring in the transition region between apex and shank, is crucial for cell shape determination (Parre and Geitmann, 2005a; Fayant et al., 2010; Palin and Geitmann, 2012). Depolymerization of short randomly-oriented MTs in the pollen tube tip by low concentrations of Noc show that MTs play a role in the fine control of tube diameter (Idilli et al., 2013). Nevertheless, how MTs are involved in this process is not yet defined. It is possible to hypothesize that MTs affect endo-exocytosis or recycling/repositioning processes that could influence PME/PMEI function in the tip (Figure 1). As reported above, MTs are involved in internalization events in the tip and regulate slow exocytosis in the central area of the apex, since presence of the MT depolymerising drug Noc inhibits endocytosis and enhances secretion speed in this zone (Moscatelli et al., 2012; Idilli et al., 2013). Alternatively or additionally, MTs could control cell wall composition by modulating pectin deposition. In *Nicotiana tabacum*, rings of de-esterified pectins are observed along pollen tubes, coinciding with pulsed growth cycles (Derksen et al., 2011). Analysis of tobacco pollen tubes by cryo-FESEM and VEC-LM suggests that during pollen tube elongation, a high rate of exocytosis in the apical flanks precedes a period of fast growth and coincides with the formation of a thick wall. During fast growth, this thick wall moves along the shank and a new thin wall forms by exocytosis at the very tip. As a consequence, ring-like areas consisting of thick de-esterified pectins alternate with thin cell wall interband regions (Derksen et al., 2011). MTs may therefore control pollen tube diameter by regulating pectin secretion and pectin esterification status in the transition zone between the apex and shank during pulsed growth.

### CELLULOSE SYNTHASE PLASMA MEMBRANE TARGETING

Besides de-esterified pectins, control of pollen tube shape and diameter has also been attributed to crystalline cellulose (Aouar et al., 2010). Cellulose microfibrils are highly resistant to tensile stress and determine the direction of cell expansion (Green, 1962). In cylindrical pollen tubes, cellulose microfibrils are oriented in a helical/longitudinal direction, nearly parallel to the longitudinal axis of the cell (Aouar et al., 2010; Chebli et al., 2012). This orientation does not control the direction of pollen tube elongation as in somatic cells, but rather confers tensile resistance in the transition areas between the tip and the cylindrical shaped regions maintaining, together with pectins, tube diameter and shape (Aouar et al., 2010; Chebli et al., 2012). Furthermore, the longitudinal microfibril arrangement sustains the pollen tube during its journey through the style transmitting tissue to the ovules (Geitmann, 2010; Chebli et al., 2012).

In somatic plant cells, co-localisation of CESA proteins and MTs suggest that MTs and CESA interact functionally: MTs play a role in CSC positioning in the PM and in stabilizing their movement during microfibril synthesis (Paredez et al., 2006). In tobacco pollen tubes, cellulose microfibrils orient in the same direction as cortical MTs in the sub-apical region and CSCs also partially align with MTs (Cai et al., 2011, 2014). However, failure to visualize MTs in live pollen tubes (Idilli et al., 2013) means that data on the direct physical interaction between CSC and MTs is not yet available. Nevertheless, it is possible to suppose that in pollen tubes, MTs could have a role in positioning CSCs in specific PM domains, as observed in somatic cells (Crowell et al., 2009; Gutierrez et al., 2009).

In *Arabidopsis* and tobacco, CESA-containing vesicles are secreted in the apical flanks of pollen tubes and CSCs are observed in shank and tip PM (Figure 1; Cai et al., 2011; Chebli et al., 2012). In *Arabidopsis*, CSCs are activated in secretory vesicles prior to their insertion into the PM. Cellulose has been observed in the tip of *lily* and *Arabidopsis* pollen tubes, where it may confer additional reinforcement (Fayant et al., 2010; Chebli et al., 2012). In tobacco, although CESA proteins is reported in the apical dome (Cai et al., 2011), crystalline cellulose is observed 5–15 μm behind the tip (Ferguson et al., 1998), suggesting that cellulose is deposited in the apex in a disorganized way and therefore does not determine the direction of growth (Guerriero et al., 2014). Immunolocalization experiments show that CESA is also present in the inverted cone region (Cai et al., 2011) where exocytic and endocytic vesicles localize (Figure 1). As reported, it is hypothesized that the clear zone represents a sorting station involved in repositioning and recycling PM proteins/lipids and in delivering apically internalized materials through the MT-dependent degradation pathway (Idilli et al., 2013). In somatic cells, CESA is observed in small vesicles which exhibit erratic movements and localize immediately behind the PM (Paredez et al., 2006). These compartments, small CESA compartments, SmaCCSs (Gutierrez et al., 2009), or MT-associated CESA compartments, MASCs (Crowell et al., 2009; Wightman and Turner, 2010) are associated with MTs and are interpreted as delivery compartments formed before their fusion to the PM (Gutierrez et al., 2009) or as intracellular stores of internalized CESA proteins (Crowell et al., 2009). In pollen tubes, cellulose synthesis is achieved differently in shank and tip, since microfibrils seem organized in the subapical region and disorganized in the tip, responding to the mechanical properties of different tube areas (Cai et al., 2011; Guerriero et al., 2014). In pollen tubes as in somatic cells, CESA activity is also regulated by internalization processes occurring in the shank and tip (Chebli et al., 2012). Mutation of the dynamin-related proteins AtDRP1A and OsDRP2B suggests that clathrin-dependent endocytosis (CDE) plays a role in cellulose biosynthesis (Collings et al., 2008; Xiong et al., 2010). Furthermore, CESA seems to be functionally associated with μ2, a subunit of an AP2 (adaptor protein complex 2) involved in the recruitment of CDE cargoes and proteins of the CDE machinery (Rohde et al., 2002; Bashline et al., 2013; Kim et al., 2013). Interaction between μ2-adaptin and CESA suggests that CSCs are internalized by CDE (Bashline et al., 2013, 2014a). In pollen tubes, CDE occurs in apical and sub-apical regions in MT-dependent and actin-dependent manners, respectively (Moscatelli et al., 2007; Idilli et al., 2012, 2013; Onelli and Moscatelli, 2013). We speculate that MTs play a role in the internalization and recycling/repositioning of CSCs in the tip by affecting endocytosis and exocytosis. In tobacco pollen tubes, recycling/repositioning of PM components in the tip appears to depend on the more dynamic MTs observed in this area (Idilli et al., 2012, 2013). It is interesting to note that SmaCCSs/MASCs are associated with cortical MTs in somatic cells and that movements of these compartments coincide with MT-depolymerizing ends (Crowell et al., 2009; Gutierrez et al., 2009). Thus, CSC regulation/movement in the pollen tube apex could depend on MT dynamics.

In somatic cells, internalized vesicles are recycled by VHA-a1 compartments (TGN; Dettmer et al., 2006; Crowell et al., 2009). In the pollen tube, CSC recycling and repositioning might involve CESA-containing vesicles of the inverted cone region, further supporting the idea that they are a crucial sorting compartment which may function as a TGN/EE (Figure 1; Viotti et al., 2010; Cai et al., 2011; Moscatelli et al., 2012; Idilli et al., 2013). To complicate the puzzle, several cellulose-synthase-like proteins (CLS), differently involved in cell wall polysaccharide synthesis, have been identified (Yin et al., 2009). Among these, CSLD1 and CSLD4 were identified in *Arabidopsis* pollen tubes and show polar localization in the tip PM. These proteins are predominantly expressed in tip-growing cells, in which low levels of CESA are detected, suggesting that CSLDs may be involved in the synthesis of cellulose in the tip region of the pollen tube (Wang et al., 2011). Their localization in the tip together with the role of MTs in tip membrane trafficking suggest that MTs may play a role in restricting these proteins to specific PM domains (Figure 1).

An additional or alternative role of MTs in defining cell wall composition may be the modulation of cellulose crystallinity in the pollen tube apex. Cell wall resistance to tensile forces and thus the direction of cell expansion appear to depend on the degree of cellulose crystallinity and microfibril length (Wasteneys and Fujita, 2006; Fujita et al., 2011, 2012). MT disruption in AT2G35630 MICROTUBULE ORGANIZATION 1 mutant (*mor-1*) alters CSC velocity and cellulose crystallinity, affecting the mechanical properties of the cell wall (Kawamura et al., 2006; Kawamura and Wasteneys, 2008; Fujita et al., 2011). Furthermore, in the tip the presence of short MT strands in the core cytoplasm produce short and mechanically weak microfibrils, resulting in loss of anisotropic expansion (Wasteneys, 2004). Short and dynamic MTs in the pollen tube apex, may modulate CSC or CLS activity or their long residence in the PM, thus affecting the structure of cellulose microfibrils in the tip and pollen tube elongation.

### MEMBRANE MICRODOMAINS AS DETERMINANTS OF CELLULOSE SYNTHASE ACTIVITY

An intriguing hypothesis suggests that cortical MTs influence PM lipid composition and fluidity. Dynamic MTs also define specific PM domains which in turn affect CSC activity and movement (Emons et al., 1992; Fujita et al., 2012). Asymmetrical PM lipid composition has an important role in polarized pollen tube growth. For example, PA accumulates in the subapical PM in growing pollen tubes (Potocký et al., 2003, 2014). The PA is implicated in regulating CDE, inducing PM curvature by changing PM lipid geometry (Pleskot et al., 2012; Potocký et al., 2014). In the apical flank, PA localization merge with lipids involved in signal transduction, such as diacyl glycerol (DAG) and phosphatidyl inositol 4,5-bisphosphate (PIP_2_; Potocký et al., 2014). Unlike PA, PIP_2_ accumulates in the tip (Kost et al., 1999) and is restricted in this area by the activity of phospholipase C localized behind the apex (Dowd et al., 2006; Helling et al., 2006). PIP_2_ plays a role in exocytosis and endocytosis at the tip (Kost et al., 1999; Zhao et al., 2010). Hydrolysis of PIP_2_ results in formation of 1,4,5-triphosphate (IP_3_) and DAG which is endocytosed and recycled to maintain its symmetrical distribution in the tube tip. These lipids are also involved in secretion, in establishing the Ca^2+^ gradient and in regulating Ca^2+^-dependent signaling (Monteiro et al., 2005). Phosphorylation of DAG by diacylglycerol kinase (DGK) forms PA which plays a critical role as signaling molecule in the regulation of tip growing pollen tubes (Pleskot et al., 2012). Phosphoinositides, DAG and PA can represent sites for the targeting of effector proteins to specific membrane domains or for modulating membrane curvature (Pokotylo et al., 2013).

Intriguingly, in root hairs PIP_2_ overlaps with a sterol-enriched PM platform that delimits the tip (Ovecka et al., 2010). In animal cells and in somatic plant cells, specific PM domains, called membrane rafts, are enriched in sterols, sphingolipids and highly saturated phospholipids and seem to interact with AFs and MTs (Zambito and Wolff, 1997, 2001; Mongrand et al., 2004; Borner et al., 2005; Lefebvre et al., 2007; Dinic et al., 2013). Rafts or detergent-resistant membranes (DRMs) recruit specific sets of proteins and may restrict cell processes in specific PM domains (Schroeder et al., 1994; Brown and London, 1997; Mongrand et al., 2004; Borner et al., 2005; Laloi et al., 2007; Lefebvre et al., 2007; Cacas et al., 2012; Muñiz and Zurzolo, 2014). The involvement of membrane microdomains in pollen tubes was defined in the gymnosperm *Picea meyeri* (Liu et al., 2009). The use of filipin and live cell imaging by di-4-ANEPPDHQ showed that sterol-enriched microdomains were polarized in the growing tip. Disruption of membrane microdomain polarization dissipates the Ca^2+^ gradient and attenuates production of tip-based NADPH oxidase (NOX)-dependent reactive oxygen species (ROS; Liu et al., 2009). The involvement of lipid microdomains rich in sterols and sphingolipids in angiosperm pollen tubes still need to be investigated.

Several mutants in sterol biosynthesis showed that cellulose formation depends on the presence of sterols in the PM (Schrick et al., 2004, 2012). In hybrid aspen and tobacco somatic cells, CESA occurs in DRMs and 85% of the CESA catalytic subunit segregates in the DRM fractions (Colombani et al., 2004; Bessueille et al., 2009). It has also been proposed that sterols in DRMs provide a primer for cellulose biosynthesis (Peng et al., 2002) or a scaffold to ensure the proper structural conformation of CESA and to stabilize CSCs (Schrick et al., 2012). In pollen tubes, DRMs containing actin and tubulin, together with proteins involved in membrane trafficking, have been identified (Moscatelli et al., 2015, paper in press). Further functional studies are necessary to elucidate the role of membrane microdomains in exo-endocytosis and in cytoskeleton dynamics. CSCs may interact with cortical MTs and lipids in specific PM domains to regulate polarized growth. Another piece of this puzzle is identification of proteins representing a possible link between CESA and MTs. One of these proteins is a glycosyl-phosphatidyl inositol (GPI)-anchored protein COBRA which is involved in controlling anisotropic expansion. GPI-anchored proteins usually contribute to raft platform stability (Schroeder et al., 1994; Paladino et al., 2004; Paulick and Bertozzi, 2008) and could be required for cell wall synthesis (Gillmor et al., 2005). Specifically, in somatic plant cells, COBRA affects cellulose microfibril orientation and crystallization in a MT-dependent manner (Li et al., 2003; Brown et al., 2005; Roudier et al., 2005; Wasteneys and Fujita, 2006; Liu et al., 2013). In *Arabidopsis* pollen tubes, COBRA_LIKE10 protein (COBL10) is localized in the apex PM and pectin and cellulose deposition are affected in *cobl10*. It is hypothesized that COBL10 could regulate the clustering of lipid rafts at the very tip of pollen tubes (Li et al., 2013).

Cellulose synthase interactive proteins (CSI1 and CSC3) are other factors playing a role in cellulose biosynthesis and anisotropic cell expansion in somatic cells (Gu et al., 2010; Gu and Somerville, 2010; Lei et al., 2013). CSI1 co-localizes with CSCs and moves along cortical MTs, suggesting a role in alignment of CSC trajectories and MTs (Gu et al., 2010; Li et al., 2011; Lei et al., 2012, 2013). Putative lipid-binding activity in the C2 domain of CSI1 suggests that this protein may act as a scaffold for CSCs, MTs and lipid rafts (Lei et al., 2014). Altogether, the various evidence supports the idea that MTs play a major role in cell wall modulation, particularly in the pollen tube apical dome, and opens new perspectives on the role of lipids and PM domains in rapid polarized cell growth.

### CALLOSE DEPOSITION

Beside pectins and cellulose microfibrils, callose also occurs in large amounts in pollen tube cell walls. Unlike cellulose, which plays an important mechanical role in the transition region between the hemispherical apex and the cylindrical shank (Parre and Geitmann, 2005b; Aouar et al., 2010), callose stabilizes and reinforces the cylindrical sub-apical region of pollen tubes (Parre and Geitmann, 2005b; Cai et al., 2011; Chebli et al., 2012). Callose deposition occurs in the shank and the plugs that isolate the growing apical cytoplasmic region from old distal vacuolated areas (Cresti and van Went, 1976; Carpita and Gibeaut, 1993; Ferguson et al., 1998). Callose has not been detected in the tip (Ferguson et al., 1998; Cai et al., 2011; Chebli et al., 2012). The application of mechanical stress to pollen tubes showed that callose is important for resistance to circumferential tensile stress (Parre and Geitmann, 2005b). Callose synthase (CalS) is a large, PM-localized complex secreted in the apical flanks by Golgi-derived vesicles and distributing in the apical and distal regions (Figure 1; Brownfield et al., 2007, 2008; Cai et al., 2011; Chebli et al., 2012). Immunofluorescence analysis and drugs affecting MT dynamics have shown that MTs are important for proper deposition of callose in the plugs and for insertion of CalS in the distal PM (Cai et al., 2011); otherwise MTs do not affect delivery and secretion of CalS in the apex (Cai et al., 2011).

Besides cellulose biosynthesis, lipid rafts also appear to be involved in callose formation in somatic cells. Detection of callose activity in isolated DRMs and biochemical characterization of glucans synthesized *in vitro* by DRMs confirm the occurrence of callose and CESAs in DRMs (Colombani et al., 2004; Bessueille et al., 2009; Morel et al., 2006). The presence of CalS in the pollen tube tip (Cai et al., 2011) where callose is absent intriguingly suggests that CalS activity could be controlled by the retrieval of the enzyme from the apical PM to be repositioned in the shank and that MTs could be involved in this process. Even if CalS has not been identified in tobacco pollen tube membrane microdomains (Moscatelli et al., 2015), further studies could better characterize DRM-proteins and investigate the role of MTs in functional relationships between membrane rafts, membrane trafficking and cell wall composition in polarized growth.

## MICROTUBULES AND POLLEN TUBE/PISTIL INTERACTION

Appropriate pollen tube growth plays a key role in conveying sperm cells to the embryo sac for double fertilization. Pollen landing on the pistil interacts with stigma papillae for adhesion, hydration and germination. Pollen tubes then travel in the transmitting tissue of the style, grow along the funiculus and finally enter the embryo sac through the micropyle. Pollen tube reception includes tip bursting and degeneration of synergids to allow sperm discharge and double fertilization (Palanivelu and Tsukamoto, 2012; Dresselhaus and Franklin-Tong, 2013). During their journey to the ovule, crosstalk between the male gametophyte and molecules of the pistil-secreted extracellular matrix (ECM) occurs to support, attract and guide pollen tubes (Higashiyama et al., 2003; Higashiyama, 2010; Takeuchi and Higashiyama, 2011; Palanivelu and Tsukamoto, 2012; Dresselhaus and Franklin-Tong, 2013; Guan et al., 2013). These interactions facilitate or prevent pollen tube elongation, inducing changes in the gene expression pattern in different stages of the pollen tube journey, both in the male gametophyte and in the female tissues (Wang et al., 2008; Qin et al., 2009; Boavida et al., 2011; Leydon et al., 2014). Little data is available on pollen tube cytoplasmic targets of crosstalk with the pistil ECM. Nevertheless, the function of endomembrane trafficking in internalization and processing style proteins and the emerging role of MTs in endo- and exocytosis (Idilli et al., 2013) open new perspectives regarding the contribution of the cytoskeleton to pollen-pistil interactions.

### INTERACTIONS PROMOTING POLLEN TUBE GROWTH

Microtubules may possibly be involved in adhesion to transmitting-tissue ECM. Adhesion is the first event occurring on the stigma, where it involves recognition processes and also plays a role in guiding the pollen tube toward the ovary (Lord, 2003; Kim et al., 2004). In lily this interaction is mediated by pectins and a stigma/stylar cysteine-rich adhesin (SCA) secreted in the ECM (Mollet et al., 2000; Park et al., 2000; Chae et al., 2007). The action of SCA in pollen tube adhesion and guidance involves interaction with the pollen tube surface and also a more intimate relation. In fact, while SCA functions as an adhesive matrix in the region behind the tip, in the tip region it binds the PM and is endocytosed and delivered to vacuoles, suggesting that it plays a role in pollen tube growth signaling (Ravindran et al., 2005; Kim et al., 2006). Interestingly, SCA is internalized by CDE and sorted to vacuoles without Golgi involvement (Kim et al., 2006). The same pattern has been observed for negative-charged nanogold (Ng^–^) internalization in the tobacco pollen tube tip by CDE (Moscatelli et al., 2007). Depolymerization of more dynamic MTs in the apex of pollen tubes by low concentrations of Noc affects Ng^–^ internalization and conveyance to degradation pathways (Idilli et al., 2013). Thus, MTs could be involved in these processes, also playing a role in signaling pathways induced by SCA protein (Figure 1).

Among the ECM proteins involved in crosstalk between pollen tubes and the pistil, arabinogalactan proteins (AGPs) play a key role both in positive and negative interactions (Castro et al., 2013; El-Tantawy et al., 2013; Losada and Herrero, 2014; Pereira et al., 2014). AGPs comprise the C2 domain-containing protein (NaPCCP) which is a transmitting tract-specific glycoprotein of *Nicotiana alata*, involved in uptake and transport of proteins from the pistil ECM to the pollen tube (Lee et al., 2009). NaPCCP has a C2 domain that binds phosphatidylinositol 3-phosphate (PI3P), which is a component of endosomes and multivesicular bodies (MVBs). PI3P is transformed into PI(3,5)P_2_ during the transition from EEs to MVBs (Zonia and Munnik, 2004). Since NaPCCP is observed in vacuole-like compartments, this protein may conceivably be involved in the internalization and processing of ECM molecules. Moreover, as MTs play a role in sorting internalized material to the degradation pathway (Idilli et al., 2013), it might be worth investigating whether NaPCCP-containing compartments interact with MTs during internalization and *en route* to vacuoles (Figure 1).

### INCOMPATIBILITY SYSTEMS

Extracellular matrix also includes secreted proteins involved in the recognition and rejection of self-pollen (Cheung and Wu, 2001; Higashiyama et al., 2001; Cheung et al., 2010; Higashiyama, 2010; Kumar and McClure, 2010; Chae and Lord, 2011; Dresselhaus and Franklin-Tong, 2013; Maruyama et al., 2013; Denninger et al., 2014; Losada and Herrero, 2014). The self-incompatibility system (SI) allows self-recognition and rejection of incompatible pollen. Angiosperms have evolved various SI mechanisms, which can be classified into two fundamentally different systems depending on taxonomy: gametophytic and sporophytic SI, GSI, and SSI respectively (Iwano and Takayama, 2012). SI is specified by S-determinant genes at a highly polymorphic, multi-allelic S-locus. In GSI, the SI phenotype is determined by the haploid genotype of the pollen (male gametophyte) and the pollen is incompatible when its S-determinant matches one of the two S-determinants expressed in the diploid pistil. In SSI, the SI phenotype of pollen is determined by the genotype of the diploid sporophyte (tapetum cells of anthers) and pollen is incompatible when both the S-determinants on the pollen surface match both pistil S-determinants. In different plant families, rejection mechanisms of incompatible pollen occur in different ways in the SSI and GSI systems (McClure and Franklin-Tong, 2006).

Evidence of involvement of pollen MTs in GSI has been observed in *Papaver* and in S-RNase-based GSI systems. In *Papaver*, the interaction between pollen and pistil S-determinants causes a rapid Ca^2+^ influx which triggers a Ca^2+^-dependent signaling cascade, inducing actin depolymerization and rapid inhibition of tube growth (Snowman et al., 2002; Huang et al., 2004; Wheeler et al., 2009, 2010; Poulter et al., 2010, 2011). Disorganization of the actin cytoskeleton in turn induces MT depolymerization, leading to programmed cell death (Poulter et al., 2008).

In S-RNase-based GSI systems, MTs are not only a target, as in *Papaver* SI, but they also participate actively in the GSI process (Meng et al., 2014). In this system, the pistil S-determinant gene encodes a glycoprotein with RNase activity (S-RNase) which is secreted in the ECM (McClure et al., 1989). This S-RNase enters the pollen tube cytoplasm where it causes degradation of RNA, arresting pollen tube growth (McClure et al., 1990). In *Petunia* and *Antirrhinum*, during compatible pollination, the non-self interaction leads to S-RNase ubiquitylation and degradation by the 26S proteasome (Zhang et al., 2009), whereas in incompatible pollination, the self-interaction does not cause S-RNase degradation and S-RNase affects the pollen tube gene expression program (Qiao et al., 2004; Sijacic et al., 2004; McClure and Franklin-Tong, 2006). An alternative model proposes that the endomembrane system plays a pivotal role in S-RNase-based GSI. In *Nicotiana* S-RNases are endocytosed both in self and non-self pollen tubes and delivered to vacuoles (Goldraij et al., 2006). Later, S-RNases remain compartmentalized in compatible pollinations while they are released into the cytoplasm by vacuole breakage in incompatible systems. Released S-RNases digest cytoplasmic RNA, causing pollen rejection (Goldraij et al., 2006).

In apple, recent *in vitro* experiments show that S-RNases are internalized and delivered to vacuoles by Golgi-derived vesicles (Meng et al., 2014). The S-RNases first accumulate in membranous compartments and are later released into the cytoplasm where they disrupt MTs, suggesting that MTs are targets of S-RNase-based GSI, as observed in *Papaver*. Interestingly, the effects of MT-depolymerising drugs or drugs affecting MT dynamics suggest that MTs also play a role in endocytosis of S-RNases: MT perturbation delays S-RNase internalization and allows incompatible pollen to grow (Meng et al., 2014). A delay in internalization is also observed when Noc is used on tobacco pollen tube tips (Idilli et al., 2013). It is postulated that tubulin polymerization facilitates PM invagination in the pollen tube tip and perturbation of MT dynamics affects internalization processes (Idilli et al., 2013). MTs may be involved in mediating internalization of factors acting in S-RNase-based GSI (Figure 1) or, more generally, in signaling patterns allowing crosstalk between pollen and pistil ECM during pollination processes.

## CONCLUSION

The involvement of MTs in organelle trafficking, endo-exocytosis, signaling and cell wall construction in pollen tubes remains elusive. Recent data supports a role of MTs in polarized growth together with new evidence of a correlation between endocytosis and exocytosis in the pollen tube apex. The emerging picture supports the idea that MTs are involved in vesicle trafficking leading to degradation pathways and in the fine delivery and recycling of proteins/lipids to specific membrane domains. This is an important feature since the proper distribution of enzymes and receptors in the PM and the spatially controlled allocation of cell wall components are essential for maintaining polarized growth and for regulating the direction of growth in style transmitting tissue.

The use of drugs affecting MT dynamics and specific probes for exo- and endocytosis has made it possible to describe MT-dependent pathways in pollen tubes. These pathways merge with those followed by proteins/lipids involved in tube elongation (PIP_2_, CESA, and SCA, as described above). These studies provide material for speculation about the role of MTs in pollen tube polarized growth and in crosstalk with style molecules, highlighting new research cues.

Furthermore, studies on involvement of the cytoskeleton in polarized growth have highlighted the close interaction between MTs and actin filaments (Zárský et al., 2009; Idilli et al., 2012; Synek et al., 2014). It can be argued that it is extremely difficult to ascribe specific roles to actin filaments or to MTs since their functions appear to be closely linked.

In somatic cells, some plant formins, which are key regulators of actin filament nucleation, also interact with MTs, suggesting that they may function to connect MTs and microfilaments in different cell processes (Deeks et al., 2010; Li et al., 2010; Bartolini and Gundersen, 2010; Wang et al., 2012a,b; Rosero et al., 2013; Breitsprecher and Goode, 2013; Qin et al., 2014). Besides formins, other proteins, as Protein 25 and the metabolic proteins homocysteine methyltransferase, phosphofructokinase, pyruvate decarboxylase, and glucan protein synthase, seem to act both on MTs and actin filaments, regulating their dynamics and functions (Ishizaki et al., 2001; Romagnoli et al., 2010), and also need to be investigated in pollen tubes.

### Conflict of Interest Statement

The authors declare that the research was conducted in the absence of any commercial or financial relationships that could be construed as a potential conflict of interest.
